# Incidental cholecystocolonic fistula in obstructive jaundice

**DOI:** 10.1002/ccr3.4510

**Published:** 2021-07-16

**Authors:** Ronald Okidi, Martin David Ogwang, Robert Natumanya, Abraham Mukalazi, Tracy Kyomuhendo, Tom Richard Okello

**Affiliations:** ^1^ Department of Surgery Lacor Hospital Gulu Uganda; ^2^ Faculty of Medicine Gulu University Gulu Uganda; ^3^ Mildmay Uganda Hospital Kampala Uganda; ^4^ Department of Surgery Lira University Lira Uganda

**Keywords:** cholecystitis, cholecystocolonic, fistula, jaundice

## Abstract

Cholecystocolonic fistula is a rare condition often diagnosed intraoperatively, requiring an adequate set of knowledge and skills to allow safe intraoperative change of prior planned surgery and alleviate significant morbidity.

## Graphical abstract

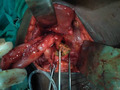

## 1 BACKGROUND

Cholecystoenteric fistula is a rare complication of gallbladder disease occurring in a minority of patients with the biliary diseases described by Courvoisier in 1890, believed to occur majorly as a result of cholecystitis.^1^, ^2^ Cholecystoduodenal fistula (70%) is the most common, followed by cholecystocolonic fistula (10%–20%).^3^

Cholecystocolonic fistula is an uncommon complication of gallbladder disease occurring in 0.06%–0.14% of patients with the biliary disease.^1^ It has also been reported in ulcerative colitis, abdominal trauma, Crohn's disease, and malignancy of the bowel, the head of the pancreas, and the biliary tract.^4^

The triad of pneumobilia, chronic diarrhea, and vitamin K malabsorption has been proposed as pathognomonic for cholecystocolonic fistula.^5^, ^6^ Although the majority of patients with cholecystocolonic fistula present with varying degree of symptoms.^7^ Extremely few cases (0.13%) have shown vague gastrointestinal symptoms which include diarrhea, abdominal pain, jaundice, nausea, vomiting, steatorrhea, and weight loss.^5^, ^8^ The presence of unabsorbed bile acids in the colon has a laxative effect causing loose stools in patients with cholecystocolonic fistula making it the most frequent symptom.^8^

The diagnosis of cholecystocolonic fistula often made intraoperatively is not easily detectable by various imaging modalities, hence the need for the surgeon to have an appropriate level of concern.^8^ Cholecystectomy, fistula resection, and a common bile duct exploration should be the treatment regardless of the clinical presentation reducing the risk of cholecystitis, cholangitis, and a 15% risk of gall bladder malignancy.^1^, ^9^ Delay in the operative management of a cholecystocolonic fistula is controversial, there are significant associated morbidity and mortality making it only an option in patients in whom its beneficial outcome outweighs the risk^10^

## 2 CLINICAL SUMMARY

N.J a 56‐year‐old female on highly active antiretroviral treatment combination of DTG/3TC/TDF for the last 6 years. She presented to the emergency department of St Mary's Hospital Lacor with abdominal pain for 6 months, associated weight loss, reduced appetite, on and off fevers, postprandial vomiting, yellowing of eyes, and itching of skin. However, she had a normal bowel and micturition habits. She had a dry cough for 2 months but no difficulty in breathing. She had no history of smoking or alcohol use. She had been undergoing management for gastritis for over 3 months.

## 3 CLINICAL EXAMINATION

She was dehydrated with mild jaundice, no conjunctival pallor, and afebrile at 36.0°C. Her blood pressure was 88/63 mm Hg with a pulse rate of 108 bpm.

The abdomen had mild right upper quadrant tenderness but with no palpable gall bladder. The respiratory, central nervous, and musculoskeletal systems examinations were unremarkable.

## 4 INVESTIGATIONS

1. An abdominal ultrasound scan showed dilated common bile duct (CBD) which measured 16.8 mm with multiple stones lodged within the lumen up to porta hepatis, biggest stone measured 34.6 × 24.7 mm. The gall bladder was well distended with echo‐free bile and no masses were seen. All other solid intraabdominal organs were normal.
2. Full hemogram revealed a normal total white cell count of 6.25 × 10^3^ cells/μl, moderate anemia (9.3 g/dl), and a normal platelet count of 460 × 10^3^ cells/μl.
3. Liver function tests had slightly elevated direct bilirubin at 0.4 mg/dl (0.0–0.3 mg/dl) and AST at 42 U/L (5–40 U/L),
4. Renal function tests: Urea and creatinine were all within a normal ranges
5. Prothrombin time 5.7 s (9–14 s), INR <0.5 (0.8–1.2)
6. Chest X‐ray was unremarkable.

## 5 MANAGEMENT

**FIGURE 1:**
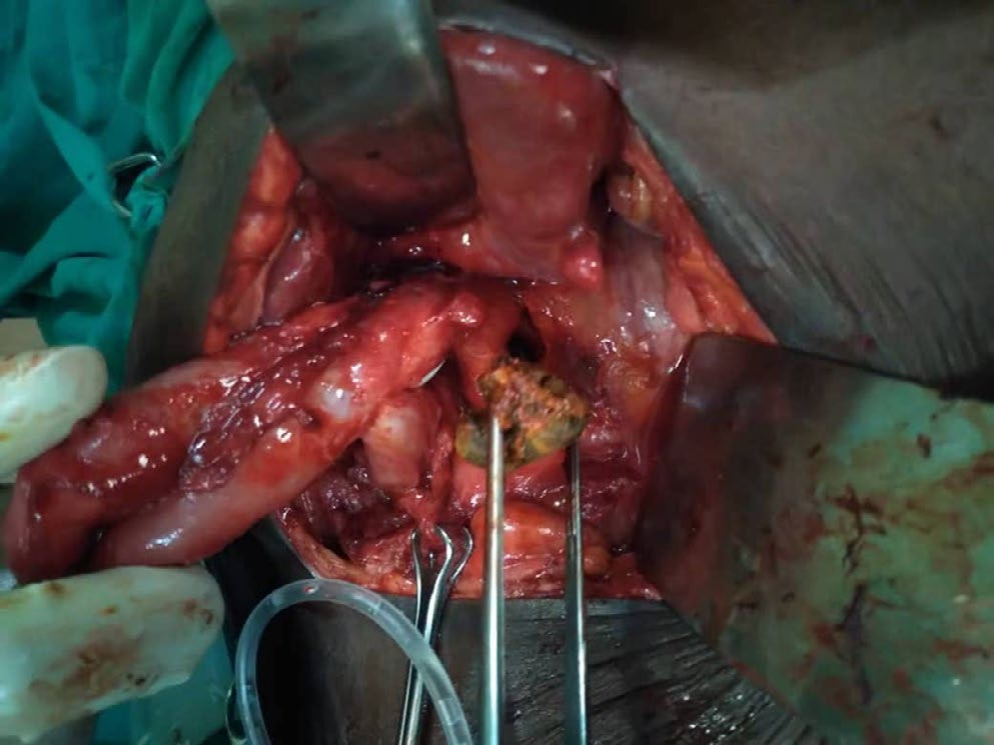
Common bile duct exploration and extraction of stones, and a cholecystocolonic fistula

She was optimized for operation with 1 unit of whole blood, 7 days course of vitamin K, and 500 ml of dextrose solution overnight and analgesia. Intraoperatively, we found multiple gallbladder stones, grossly dilated CBD containing multiple stones, and a cholecystocolonic fistula (Figure 1). Carefully performed a cholecystectomy with fistula tract resection en bloc, choledochotomy, stones extraction, choledocho‐duodenostomy, and colonic defect primary repair. Post‐operatively, she was on intravenous piperacillin‐tazobactam 4.5 grams 8 hourly for 5 days, transfused with 1 unit of whole blood and intravenous fluid maintenance therapy. She had bowel motion and started feeding normally on the 3^rd^ post‐operative day and discharged with no complication on the 9^th^ post‐operative day.

## 6 CASE DISCUSSION

Cholecystocolonic fistula (CCF) is a late complication of long‐lasting gallstone disease, and it has also been reported as a consequence of cholecystitis, trauma, peptic ulcers, cancer, diverticulitis, and advanced age.^11^ With advancing age and repeated bouts of calculus cholecystitis, in this case, there existed a high possibility of such a complication though not demonstrated by the sonogram. However, clinically suggested by the disparity in the level of jaundice not matched by the radiological findings.^1^, ^2^, ^11^ CCF seen in the advanced aged is attributable to loss of vascular smooth muscle integrity of the colon or direct damage by the direct pressure by the gallstones with spillage of bile into the colon causing an inflammatory process leading to maturation of the fistula tract.^12^ Probably, the direct pressure of the gallstone on to the gallbladder wall and the transverse colon coupled with gallbladder wall inflammation was the most likely etiology of fistulation in this patient.

The pathognomonic triad was not appreciable in her clinical presentation despite having an enterobill\iary fistula.^6^

A partial obstruction by external compression of the common bile duct and a normal distal common bile duct dimension are anatomic features frequently associated with Mirizzi's Syndrome which were not present in this patient. The presence of gallstones is easily demonstrated by noninvasive diagnostic approaches. A transabdominal US has a sensitivity of 96% regarding gallstones detection, a magnetic resonance cholangiopancreatography (MRCP) has better diagnostic accuracy and provides better information of the anatomy of the biliary tree and the gallbladder.^13^, ^14^ In our setting, MRCP was not feasible due to the low‐resource setup nonetheless a percutaneous cholangiography could have been done if the etiology of the jaundice was not appreciable by ultrasonography though missed a fistulation. Preoperative studies normally include an ultrasound scan, computed tomography scan, magnetic resonance imaging, endoscopic retrograde cholangiopancreatography, and barium enema in a well‐resourced setup. However, no single imaging modality has been proven to be highly sensitive in diagnosing a CCF,^7^ and thus, preoperative diagnosis and operative planning remain a challenge.^8^, ^15^ At surgery, a cholecystocolonic fistulation was incidentally found compelling additional surgical procedure be performed on the patient at the expertise of the surgeon.^5^, ^15^, ^16^

## 7 CONCLUSION

Cholecystocolonic fistula preoperative diagnosis is still a challenge in our setting though was suggested by unmatched clinical jaundice with the degree of common bile duct obstruction. This requires the operating surgeon to have vast knowledge and skills on the various options in the surgical management of hepatobiliary pathologies.

## CONFLICT OF INTEREST

None declared.

## AUTHOR CONTRIBUTIONS

All authors actively participated in writing the manuscript and discussion of the case. RO operated and was involved in the post‐operative care of the patient until discharge.

## ETHICAL APPROVAL

Ethical approval was obtained from Lacor Hospital Institutional Research and Ethics committee (LHIREC) and the hospital administration issued clearance to report this case.

## CONSENT

The patient consented to the publication of her case.

## Data Availability

The data for cholecystocolonic fistula in surgical jaundice can be found in St. Mary's Hospital.
